# Fibroblast-Derived Induced Pluripotent Stem Cells Show No Common Retroviral Vector Insertions

**DOI:** 10.1634/stemcells.2008-0696

**Published:** 2009-02

**Authors:** Florencio Varas, Matthias Stadtfeld, Luisa de Andres-Aguayo, Nimet Maherali, Alessandro di Tullio, Lorena Pantano, Cedric Notredame, Konrad Hochedlinger, Thomas Graf

**Affiliations:** a Differentiation and Cancer, Center for Genomic Regulation and Pompeu Fabra UniversityBarcelona, Spain; b Cancer Center and Center for Regenerative Medicine, Massachusetts General Hospital, Harvard Stem Cell InstituteBoston, Massachusetts, USA; c Bioinformatics Program, Center for Genomic Regulation and Pompeu Fabra UniversityBarcelona, Spain; d Institució Catalana de Recerca i Estudis Avançats

**Keywords:** Induced pluripotent stem cells, Retroviral insertions, Cellular reprogramming, Retroviral tagged mouse genes, Insertional mutagenesis

## Abstract

Several laboratories have reported the reprogramming of mouse and human fibroblasts into pluripotent cells, using retroviruses carrying the *Oct4*, *Sox2*, *Klf4*, and *c-Myc* transcription factor genes. In these experiments the frequency of reprogramming was lower than 0.1% of the infected cells, raising the possibility that additional events are required to induce reprogramming, such as activation of genes triggered by retroviral insertions. We have therefore determined by ligation-mediated polymerase chain reaction (LM-PCR) the retroviral insertion sites in six induced pluripotent stem (iPS) cell clones derived from mouse fibroblasts. Seventy-nine insertion sites were assigned to a single mouse genome location. Thirty-five of these mapped to gene transcription units, whereas 29 insertions landed within 10 kilobases of transcription start sites. No common insertion site was detected among the iPS clones studied. Moreover, bioinformatics analyses revealed no enrichment of a specific gene function, network, or pathway among genes targeted by retroviral insertions. We conclude that Oct4, Sox2, Klf4, and c-Myc are sufficient to promote fibroblast-to-iPS cell reprogramming and propose that the observed low reprogramming frequencies may have alternative explanations.

## INTRODUCTION

A major goal of current stem cell research is the use of specialized cells obtained from patient-derived embryonic stem (ES) for therapeutic purposes. A giant leap closer to this goal was made with the discovery that the expression of the transcription factors Oct4, Sox2, Klf4, and c-Myc induced the reprogramming of mouse skin-derived fibroblasts into induced pluripotent stem (iPS) cells capable of differentiating into cells of all three germ layers in teratomas and viable chimeric mice [1]. Similar results were also reported from other laboratories [2, 3] and for human cells [4]. Common to these studies is the low frequency of cell reprogramming, estimated to be approximately 0.1% or less in all cases. The reason for the observed low frequencies is not clear, but it is possible that additional genes have to be activated by insertion of retroviral vectors [5].

Integration sites of gammaretroviruses such as Moloney leukemia virus are known to be biased toward transcription start sites of actively transcribed genes [6, 7]. The retroviral long terminal repeats (LTR) act as promoter/enhancer elements that can modulate the expression of adjacent genes, frequently leading to their upregulation [8]. On the basis of repeated detections of specific genes targeted by integration in independently derived tumors from retrovirus infected mice, viral insertion strategies have been used to identify proto-oncogenes [9]. More recently this strategy was also successful in identifying genes that help to expand the hematopoietic stem cell pool [10, 11].

A recent report concluded that liver- and stomach-derived iPS cells exhibit no common retroviral integrations, suggesting that expression of Oct4, Sox2, Klf4, and c-Myc is sufficient for reprogramming [12]. However, since these cell types exhibited only 4–6 integration sites per clone, it is still possible that fibroblasts, in which a larger number of integration sites are typically observed, differ in this respect. Therefore, to explore whether fibroblast reprogramming requires the activation of additional host genes by retroviral insertion, we exhaustively determined the integration sites in six iPS clones obtained from mouse embryo- and tail tip-derived fibroblasts after infection with retroviral vectors encoding the four Yamanaka transcription factors. We identified and sequenced 93 retroviral insertion sites and mapped 79 insertions to a single location in the mouse genome. No evidence was obtained for an insertion site common to several or all clones, nor was any gene function, gene network, or canonical pathway preferentially associated with the targeted genes. Our data therefore indicate that Oct4, Sox2, Klf4, and Myc are sufficient to induce iPS cell reprogramming in fibroblasts, extending the conclusion reached by Aoi et al. with liver- and stomach-derived cells [12].

## MATERIALS AND METHODS

### Generation of iPS Cell Clones

Stable iPS cell lines were established as previously described [3]. Briefly, fibroblast cultures were established from postnatal tail-tip biopsies (for iPS clones A, B, C, and F) or from E14.5 mouse embryos (iPS clones D and E). cDNAs for murine Oct4, Sox2, and Klf4, as well as human c-Myc (the constitutively active T58A mutant), were cloned into the retroviral pMX vector and transfected into PlatE packaging cells cultured in standard ES medium (Dulbecco's modified Eagle's medium supplemented with 15% fetal bovine serum, nonessential amino acids, L-glutamine, penicillin-streptomycin, β-mercaptoethanol, and 1,000 U/ml leukemia inhibitory factor) on 15-cm plates using Fugene reagent (Roche Applied Science, Basel, Switzerland, https://www.roche-applied-science.com). Viral supernatant was harvested at 24, 48, 72, and 96h after transfection, filtered using a 0.45-μm filter, supplemented with 5 μg/ml polybrene, and added to the respective fibroblasts cultured on 10-cm plates. Colonies with iPS-like morphology were picked 3–5 weeks later and expanded on irradiated feeder cells in ES medium.

### Retroviral Insertion Sites into iPS Cell Clones Quantified by Southern Blotting

Ten micrograms of genomic DNA of each of the six iPS lines, as well as of V6.5 ES cells, was digested with BglII (for c-Myc), BamHI (for Klf4 and Oct4), and HindIII (for Sox2) and separated on a 0.8% agarose gel. DNA was blotted onto HybondXL membrane (Amersham Biosciences, Piscataway, NJ, http://www.amersham.com) and hybridized with the respective cDNA probes labeled with [^32^P]α-dCTP as described [3].

### Mapping of Retroviral Insertion Sites into iPS Clones

Retroviral insertion sites into iPS were identified by sequencing the junction fragments between the proviral 5′LTR and the host mouse genome, amplified by LM-PCR essentially as previously reported [13]. Briefly, DNA extracted from each iPS clone was digested overnight at 65°C with the frequent cutter restriction enzyme Tsp509I (Fermentas Life Sciences, Burlington, ON, Canada, http://www.fermentas.com), which recognizes the tetranucleotide AATT as its target sequence. The biotin-labeled and LTR-hybridizing primer LTR-Irev (AGCTGTTCCATCTGTTCTTGGCCCT) was annealed to the digested DNA and extended with the proofreading Pfu DNA polymerase (Stratagene, La Jolla, CA, http://www.stratagene.com). The resulting biotin-tagged double-stranded DNA fragments were recovered with a magnet after incubating with streptavidin-coated magnetic beads (Dynabeads M280; Invitrogen, Carlsbad, CA, http://www.invitrogen.com). An asymmetric linker cassette (LC) resulting from annealing LC1 (GACCCGGGAGATCTGAATTCAGTGGCACAG) and LC2 (CTGTGCCACTG) oligonucleotides was attached with T4 DNA ligase (Fermentas) to the biotin-free edge, the resulting DNA was denatured with 0.1 N NaOH, and single-stranded biotin-labeled DNA was recovered. Afterward, a first polymerase chain reaction (PCR) (first-step LM-PCR) was performed with primers LTR-IIrev (GACCTTGATCTGAACTTCTC) and LCPCR1for (GACCCGGGAGATCTGAATTC), followed by a nested PCR (second-step LM-PCR) with LTR-IIIrev (TCCATGCCTTGCAAAATGGC) and LC-PCR2for (GATCTGAATTCAGTGGCACAG). An LM-PCR internal control was provided by a 278-base pair (bp) amplicon resulting from the 3′LTR located LTR-IIIrev primer up to a retroviral vector backbone carried Tsp509I target sequence. The positioning for all LM-PCR-related primers on the resulting amplicons is shown in supporting information Figure 2. Amplicons for each iPS DNA sample were shortly electrophoresed in a 2% agarose gel and excised in three regions, corresponding to high, medium, and low molecular weight (supporting information Fig. 3). Double-stranded DNA from these three regions was separately extracted with MinElute columns (Qiagen, Germantown, MD, http://www1.qiagen.com) and directly cloned for sequencing into pGEM-T Easy Vector (Promega, Madison, WI, http://www.promega.com). A preliminary PCR-based screen on individually grown bacterial colonies was performed using T7 and SP6 primers that anneal to the cloning plasmid. Amplicons were electrophoresed in 1% agarose gels, and their sizes were determined with the Quantity One software (Bio-Rad, Hercules, CA, http://www.bio-rad.com) using a Molecular Imager Gel Doc XR System (Bio-Rad). Amplicons differing by more than 5 bp were considered potentially different and sequenced.

To increase coverage of retroviral insertions, amplicons were directly sequenced by 454 Genome Sequencer (FLX System; Roche Applied Science). With that goal, the nested primers for the second-step LM-PCR described above were replaced by the primers A-LCPCR2for and B-code-LTR-IVrev, respectively. Primer A-LCPCR2for (*GCCTCCCTCGCGCCATCAG*GATCTGAATTCAGTGGCACAG) consisted of Roche's primer A (19-mer, shown in italics) as required for the high-throughput sequencing, fused to LCPCR2for. On the other hand, the primer B-code-LTR-IVrev (*GCCTTGCCAGCCCGCTCAG*NNNNGCTTGCCAAACCTACAGGTG) results from Roche's primer B (19-mer, shown in italics) fused to a 4-mer oligonucleotide tag (shown as underlined N) plus a final LTR-IVrev primer annealing up to 10 nucleotides apart from the 5′LTR edge. The tags correspond to the sequences GATC, CGAT, TCGA, ATCG, AACG, and CGAA for the A, B, C, D, E, and F iPS clones, respectively. These tags acts as code bars that enable unequivocal tracking of the iPS clone contributing each single amplicon and so allow mixing of the second-step LM-PCR products from the six iPS clones. This amplicon mix was analyzed by high-throughput sequencing (Lifesequencing S.L., Paterna, Spain, http://www.lifesequencing.com) from primer B-code-LTR-IVrev. The sequences obtained were then processed to determine the different sequences and their relative abundances.

Amplicons sequenced either after cloning in bacteria or by high throughput were analyzed for homologies using a publicly available mouse genome sequence data-base (Ensembl BLAST [http://www.ensembl.org/Multi/blastview]; Ensembl release 49, March 2008). Insertion sites obtained by bacterial cloning consisted of amplicons containing mouse genome sequences flanked by the viral sequence from the LTR-IIIrev primer up to a CA dinucleotide and the linker cassette primer LCPCR2for joined by the tetranucleotide AATT (supporting information Fig. 2). High-throughput sequencing of amplicons was performed only from B-code-LTR-IVrev primer. In addition, short amplicons enabled to read even the complementary sequence to the primer A-LCPCR2for. Eighty-one percent of the sequences contained the expected AATT linker, with 13% replacing AATT with AATC, and 1%–3% carried AACT, CACT, or CCCA sequences. We defined genuine integration sites as those showing at least 95% sequence homology with the annotated mouse genome and matching one single genomic locus [6]. The precise location of the retroviral insertions was defined as the first nucleotide at the junction between the mouse genome sequence and the proviral 5′LTR.

### Gene Network Analysis for Retroviral Insertion Tagged Genes

The retroviral insertions were considered to hit gene transcription units when they landed between the transcription start and stop sites. Transcription units were considered only when included in the Refseq database (http://www.ncbi.nlm.nih.gov/RefSeq) or when they were Ensembl-known transcripts (http://www.ensembl.org). The retroviral targeted genes included those in which insertions landed in the transcription unit, as well as those with a transcription start site closest to the retroviral insertions. The tagged genes were studied using the Ingenuity Pathway Analysis software (Ingenuity Systems, Redwood City, CA, http://www.ingenuity.com) to uncover enrichments in shared gene functions, pathways, or networks. Details on the symbols used for the gene network and the connections as shown in supporting information Figure 6 can be found at https://analysis.ingenuity.com/pa/info/help/help.htm#legend.htm. To evaluate the relevance of our findings, the same software was also applied to 50 randomly selected groups of mouse genes listed in the Refseq database. To that end, all genes were arbitrarily assigned numbers and groups of random numbers selected using the Random Integer Generator software (Random.org, Dublin, Ireland, http://www.random.org/integers).

## RESULTS

### Mapping of Retroviral Insertion Sites in iPS Clones Reveals No Common Targeted Genes

To test whether there are recurrent integration sites in individually derived iPS clones generated with retroviral vectors that might point to as yet unknown collaborating reprogramming factors, we determined the integration sites of six different iPS clones derived from murine fibroblasts. Four of these clones (A, B, C, and D) have previously been determined to be pluripotent by the formation of teratomas and to generate chimeras after injection into blastocysts [3]. The other two clones (E and F) also form teratomas and differentiate into distinct mature cell types in vitro but have not been tested by blastocyst injection (M. Stadtfeld and K. Hochedlinger, unpublished data). We first performed Southern blot analyses with probes against the transcription factors Oct4, Sox2, Klf4, and c-Myc on genomic DNA extracted from the different iPS clones. The resulting hybridization bands (supporting information Fig. 1) enabled us to estimate 6–18 integration sites per iPS clone.

**Figure 1:**
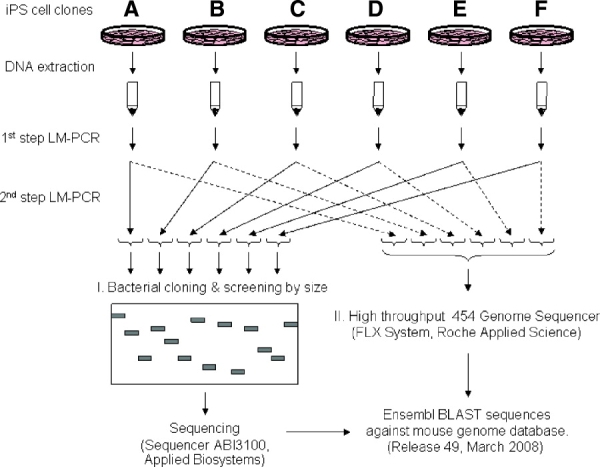
Experimental strategy for mapping retroviral insertion sites in iPS clones. DNA was extracted from individual iPS cell clones, and the fragments between the retroviral 5′ long terminal repeats and mouse genomic DNA were amplified by LM-PCR. The resulting amplicons were then sequenced either after cloning in bacterial plasmids or directly by high-throughput sequencing. Retroviral insertions were then determined by performing Ensembl BLAST searches against a mouse genome database. Abbreviations: iPS, induced pluripotent stem; LM-PCR, ligation-mediated polymerase chain reaction.

To identify and map the retroviral insertion sites we used two strategies (Fig. 1). In both approaches we first amplified the junction fragments between proviral and mouse genomic sequences by LM-PCR. The resulting amplicons were then either cloned into the bacterial plasmid pGEM-T Easy vector followed by sequencing or directly subjected to high-throughput amplicon sequencing with the 454 Genome Sequencer (FLX System; Roche Applied Science). Bacterial inserts were run on gels and screened by size (supporting information Fig. 3). Screening more than 600 bacterial colonies allowed the sequencing of 8, 7, 11, 9, 9, and 14 different junction fragments for iPS clones A–F, respectively. The coverage of insertion sites relative to those estimated by Southern blotting was below 80%, probably because of the predominant amplification of internal control bands and the heterogeneous PCR amplification efficiency of different insertions.

The high-throughput sequencing yielded 19,103 sequences that clustered into 93 amplicons. Every insertion identified by bacterial cloning was also contained in these amplicons. The retroviral insertions so identified raised the numbers of insertions per clone to 16, 12, 14, 13, 11, and 27 sites for clones A–F, respectively. Seventy-nine of those 93 amplicons/insertion sites could be mapped to specific mouse chromosomes (Table 1). Unmapped insertions correspond to sequences that aligned with more than one genomic locus, were too short to be mapped, or fell into incompletely sequenced genomic stretches.

**Table 1:** Retroviral insertion sites in fibroblast derived iPS clones

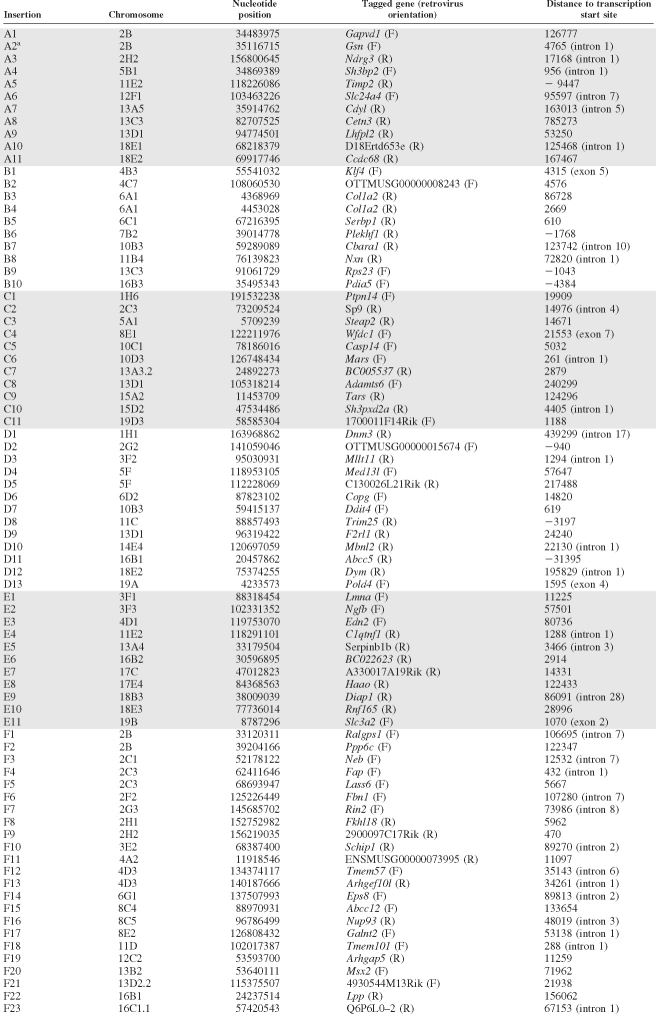

The table lists retroviral insertions found in the six iPS clones (A to F) examined. Underlined insertions were found both by bacterial sequencing and by high throughput sequencing. Genes shown in italics were included in the Ingenuity Pathway Analysis. The orientation of retroviral vector transcription was designated as forward (F) when it proceeded in the same sense than the tagged gene and reverse (R) when it pointed in the opposite direction. The distance between the retroviral insertion site and the transcription start site of the closest gene is shown in the last column. Insertions that occurred within transcription units and their locations are shown in brackets.

**Figure 2:**
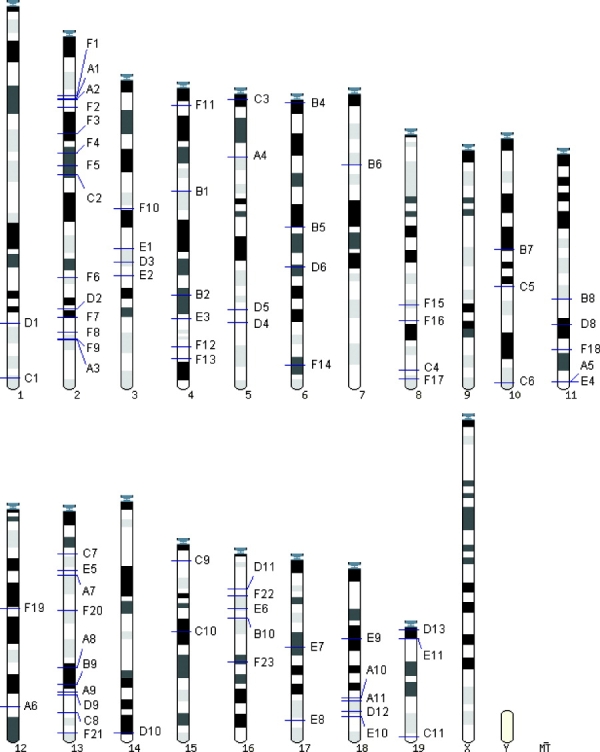
Distribution of retroviral insertion sites among mouse chromosomes. The horizontal lines across the chromosomes indicate sites of insertions.

The retroviral insertion sites were widely distributed throughout the mouse genome (Fig. 2). Eighteen of the autosomal chromosomes contained at least one insertion, whereas chromosome 9 and both sex chromosomes had no insertions. No relationship was observed between the chromosomal length and the number of retroviral insertions per chromosome. However, the insertions frequently hit the same chromosomes more than once. This is exemplified by iPS clone F, where 17 of the 23 insertions mapped were restricted to only four chromosomes (9, 3, 3, and 2 insertions in chromosomes 2, 4, 8, and 16, respectively). Most insertions (Table 1) landed in gene-rich areas, with 78% of the insertions located within 100 kilobases (kb) from the closest transcription start site. Four insertions mapped to exons and 31 to introns, with approximately half of the latter being located in the first two introns. Another 13 and 16 insertions were within 10 kb downstream and upstream of the transcription start site, respectively.

Thirty of the 79 mapped retroviral insertions in iPS clones are included in the mouse Retrovirus Tagged Cancer Gene Database (http://rtcgd.abcc.ncifcrf.gov). Importantly, our data showed that not a single gene was shared by more than one iPS clone. The two closest insertions identified (B3 and B4) were located 84 kb apart, upstream of the transcription start site for gene Col1a2 on chromosome 6. However, both were found in the same clone. We therefore conclude that no gene was targeted more than once by retroviral insertions in the different iPS clones studied, suggesting that Oct4, Sox2, Klf4, and c-Myc are sufficient to trigger fibroblast reprogramming into iPS cells.

### No Evidence That Genes Tagged by Retroviral Insertions Have Shared Gene Functions or Belong to Common Canonical Pathways or Gene Networks

**Figure 3:**
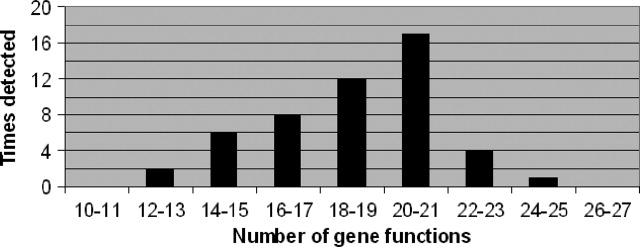
Distribution of the number of gene functions included in the best gene networks of 50 groups of 65 randomly selected genes plus *Oct4*, *Sox2*, *Klf4*, and *c-Myc*.

Although no common tagged gene was uncovered by mapping the insertion sites in iPS cells, it is still possible that some of the insertions preferentially affect specific gene functions, gene networks or signaling pathways. To determine whether retrovirally tagged genes fall into functional gene categories, the genes listed in Table 1 were grouped together with Oct4, Sox2, Klf4, and c-Myc and subjected to bioinformatics analyses using Ingenuity Pathway Analysis (Ingenuity Systems). The total number of genes so analyzed was reduced from 79 to 69 since insertions B1 and B4 were left out (they correspond to the endogenous Klf4 gene and to Col1a2, which was detected twice), and another 12 genes were subtracted because not enough biological information was available. The remaining 69 genes showed no evident enrichment for any specific gene function (supporting information Fig. 4). Similarly, no bias toward any canonical gene pathway was observed (supporting information Fig. 5). Finally, the network analysis revealed 18 genes as part of a best gene network that was enriched in components of nervous system development and function, cell cycle and cellular development (supporting information Fig. 6). To assess whether this network might be biologically relevant or a product of chance, the Ingenuity Pathway Analysis was repeated 50 times with different groups of 65 randomly selected genes plus the four Yamanaka transcription factors. This analysis revealed that the number of genes participating in best networks ranged between 13 and 28 (Fig. 3). The value obtained with the targeted genes from Table 1, 18 gene functions, therefore falls well within the range of values obtained with the randomized approach. We conclude that the retroviral insertions in the six iPS clones studied have no shared gene functions, nor do they belong to a common canonical pathway or gene network.

## DISCUSSION

Our results show that no genes tagged by retroviral insertions were repeatedly detected in the six iPS clones analyzed. In addition, bioinformatics analyses showed no enrichment of tagged genes that are functionally related or are part of the same pathways or networks. On the basis of the estimated numbers of integrations by Southern blotting, bacterial cloning and sequencing, and high-throughput sequencing (summarized in Table 2), the insertion site coverage for the six iPS clones analyzed appears to be complete. This is reinforced by the fact that the minimum frequency with which each insertion was sequenced by high throughput was 3–26 times per amplicon per clone. However, it is possible that the number of integrations detected using the Tsp509I restriction enzyme to generate the samples used for high-throughput sequencing is not exhaustive. Thus, it has been reported that the restriction enzyme used for genomic DNA digestion preceding PCR-based protocols influences the coverage of retroviral insertion sites detected [14].

**Table 2:** Retroviral insertion numbers determined by different methods

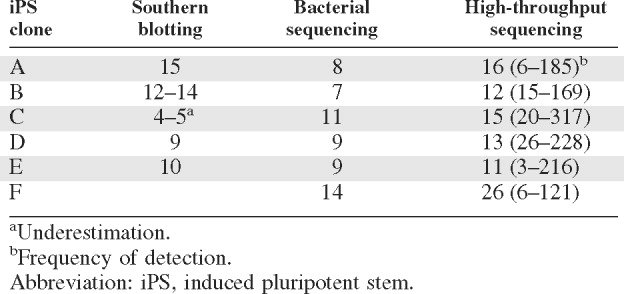

Twelve of the 28 retrovirally tagged genes previously reported in liver and hepatic-derived iPS [12] are included in the Retrovirus Tagged Cancer Gene Database [15]. Similarly, we found that an elevated percentage (38%) of the tagged genes in fibroblast-derived iPS is contained in this database, as three sets of 69 random control genes yielded 20%. This might represent a bias of integrations into actively transcribed genes, although a biological relevance of the enriched genes cannot be excluded. Importantly, bioinformatic analyses of our data showed no enrichment of tagged genes that are functionally related or are part of the same pathway or network. In addition, we found no overlap between the genes targeted in our study and those reported by another group [12]. Our results therefore indicate that Oct4, Sox2, Klf4, and c-Myc are sufficient to induce the reprogramming of fibroblasts into iPS cells and extend the observations of Aoi et al. [12] to one of the most commonly used and easily available cell types used in reprogramming studies.

There are several alternative explanations for the observed low frequencies of reprogramming. One possibility is that the cell type of origin dictates the frequency and that fibroblasts are heterogeneous, being composed of a few highly susceptible and many resistant cell types. This is unlikely, since it has been shown that epithelial cells derived from stomach, liver, and pancreatic β cells can be reprogrammed by the four Yamanaka factors, at frequencies similar to or above those seen with fibroblasts [12, 16]. However, reprogramming of mature B cells required an additional transcription factor [17]. Another possibility is that the stoichiometry of the Yamanaka factors expressed after infection is not adequate in most cells, leading to no or incomplete reprogramming, growth disadvantage, or death.

The most likely scenario, therefore, is that only a small proportion of somatic cells have a chromatin configuration amenable to transcription factor-induced changes resulting in iPS cell formation. Two lines of evidence support this notion. First, daughter cells derived from a partially reprogrammed iPS cell clone have been shown to reactivate Oct4 at different times, consistent with the involvement of stochastic epigenetic events leading to the reacquisition of pluripotency [18]. Second, the treatment of partially reprogrammed iPS cells or of somatic cells undergoing reprogramming with compounds targeting epigenetic modifications, including DNA and histone methylation as well as histone acetylation, improves the efficiency and fidelity of reprogramming significantly [19–21].

## CONCLUSION

The analysis of six fibroblast-derived iPS cell clones induced after infection with retroviruses expressing Oct4, Sox2, Klf4, and c-Myc showed no evidence of common retroviral integration sites. Taking these data together with supporting bioinformatics analyses, we suggest that the activation of additional endogenous genes is not required for the reprogramming induced by the four factors. It will now be interesting to see whether the four Yamanaka factors are sufficient to generate viral integration-free iPS cells for research and regenerative medicine.

## NOTE ADDED IN PROOF

Two communications have now shown that the formation of iPS cells can be generated without using retroviruses as vectors and in the absence of detectable integrations:

Okita K, Nakagawa M, Hyenjong H, Ichisaka T, Yamanaka S. Generation of mouse induced pluripotent stem cells without viral vectors. Science. 2008;322:949–953.

Stadtfeld M, Nagaya M, Utikal J, Weir G, Hochedlinger K. Induced pluripotent stem cells generated without viral integration. Science. 2008;322:945–949.

## References

[1] Okita K, Ichisaka T, Yamanaka S (2007). Generation of germline-competent induced pluripotent stem cells. Nature.

[2] Wernig M, Meissner A, Foreman R (2007). In vitro reprogramming of fibroblasts into a pluripotent ES-cell-like state. Nature.

[3] Maherali N, Sridharan R, Xie W (2007). Directly reprogrammed fibroblasts show global epigenetic remodeling and widespread tissue contribution. Cell Stem Cell.

[4] Takahashi K, Tanabe K, Ohnuki M (2007). Induction of pluripotent stem cells from adult human fibroblasts by defined factors. Cell.

[5] Yamanaka S (2007). Strategies and new developments in the generation of patient-specific pluripotent stem cells. Cell Stem Cell.

[6] Wu X, Li Y, Crise B (2003). Transcription start regions in the human genome are favored targets for MLV integration. Science.

[7] Mitchell RS, Beitzel BF, Schroder AR (2004). Retroviral DNA integration: ASLV, HIV, and Mlv show distinct target site preferences. PLoS Biol.

[8] Mikkers H, Berns A (2003). Retroviral insertional mutagenesis: Tagging cancer pathways. Adv Cancer Res.

[9] Akagi K, Suzuki T, Stephens RM (2004). RTCGD: Retroviral tagged cancer gene database. Nucleic Acids Res.

[10] Kustikova O, Fehse B, Modlich U (2005). Clonal dominance of hematopoietic stem cells triggered by retroviral gene marking. Science.

[11] Kustikova OS, Geiger H, Li Z (2007). Retroviral vector insertion sites associated with dominant hematopoietic clones mark “stemness” pathways. Blood.

[12] Aoi T, Yae K, Nakagawa M (2008). Generation of pluripotent stem cells from adult mouse liver and stomach cells. Science.

[13] Schmidt M, Hoffmann G, Wissler M (2001). Detection and direct genomic sequencing of multiple rare unknown flanking DNA in highly complex samples. Hum Gene Ther.

[14] Wang GP, Garrigue A, Ciuffi A (2008). DNA bar coding and pyrosequencing to analyze adverse events in therapeutic gene transfer. Nucleic Acids Res.

[15] Hawley RG (2008). Does retroviral insertional mutagenesis play a role in the generation of induced pluripotent stem cells?. Mol Ther.

[16] Stadtfeld M, Brennand K, Hochedlinger K (2008). Reprogramming of pancreatic Beta cells into induced pluripotent stem cells. Curr Biol.

[17] Hanna J, Markoulaki S, Schorderet P (2008). Direct reprogramming of terminally differentiated mature B lymphocytes to pluripotency. Cell.

[18] Meissner A, Wernig M, Jaenisch R (2007). Direct reprogramming of genetically unmodified fibroblasts into pluripotent stem cells. Nat Biotechnol.

[19] Shi Y, Do JT, Desponts C (2008). A combined chemical and genetic approach for the generation of induced pluripotent stem cells. Cell Stem Cell.

[20] Huangfu D, Maehr R, Guo W (2008). Induction of pluripotent stem cells by defined factors is greatly improved by small-molecule compounds. Nat Biotechnol.

[21] Mikkelsen TS, Hanna J (2008). Dissecting direct reprogramming through integrative genomic analysis. Nature.

